# DeepSecMS Advances DIA‐Based Selenoproteome Profiling Through Cys‐to‐Sec Proxy Training

**DOI:** 10.1002/advs.202504109

**Published:** 2025-07-22

**Authors:** Chenfang Si, Yamei Yuan, Yu Zong, Liang Qiao, Wen‐Feng Zeng, Yaoyang Zhang

**Affiliations:** ^1^ Interdisciplinary Research Center on Biology and Chemistry Shanghai Institute of Organic Chemistry Chinese Academy of Sciences 100 Haike Rd. Shanghai 201210 China; ^2^ University of Chinese Academy of Sciences Beijing 100049 China; ^3^ Department of Chemistry Fudan University Shanghai 200000 China; ^4^ Center for Infectious Disease Research & School of Engineering Westlake University Hangzhou 310024 China; ^5^ Shanghai Key Laboratory of Aging Studies Chinese Academy of Sciences 100 Haike Rd. Shanghai 201210 China; ^6^ Present address: The Core Facility Center CAS Center for Excellence in Molecular Plant Sciences Institute of Plant Physiology and Ecology Chinese Academy of Sciences Shanghai 200032 China

**Keywords:** data independent acquisition, deep learning, SecMS, selenocysteine, selenoprotein

## Abstract

Selenoproteins, defined as proteins containing the 21st amino acid, selenocysteine (Sec, U), are functionally important but rare, with only 25 selenoproteins characterized in the entire human proteome to date. To comprehensively analyze selenoproteomes, previously developed selenocysteine‐specific mass spectrometry (SecMS) and the selenocysteine insertion sequence (SECIS)‐independent selenoprotein database (SIS) have provided effective tools for analyzing the selenoproteome and, more importantly, hold the potential to uncover new selenoproteins. In this study, a deep learning approach is employed to develop the DeepSecMS method. Given the rarity of Sec and its chemical similarity to cysteine (Cys, C), a proxy training strategy is utilized using a large dataset of Cys‐containing peptides to generate a large‐scale theoretical library of Sec‐containing peptides. It is shown that DeepSecMS enables the accurate prediction of critical features of Sec‐containing peptides, including MS2, retention time (RT), and ion mobility (IM). By integrating DeepSecMS with data‐independent acquisition (DIA) methods, the identification of known selenoproteins is significantly enhanced across diverse cell types and tissues. More importantly, it facilitates the identification of numerous highly scored, potential novel selenoproteins. These findings highlight the powerful potential of DeepSecMS in advancing selenoprotein research. Moreover, the proxy training strategy may be extended to the analysis of other rare post‐translational modifications.

## Graphical abstract

DeepSecMS, a deep learning platform leveraging a Cys‐to‐Sec proxy training strategy, enhances the identification of known selenoproteins and holds promise for discovering novel candidates. This proxy approach overcomes limitations associated with the rarity of known selenoproteins and demonstrates broad applicability in studying other rare protein variants and post‐translational modifications, offering a scalable framework to advance proteomic research on low‐frequency events.

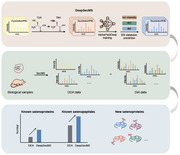

## 1 Introduction

Selenocysteine (Sec, U), often referred to as the 21st proteogenic amino acid, is a cysteine (Cys, C) analogue that contains a selenol group instead of a thiol group. This selenol group exhibits higher reactivity than the thiol, making numerous selenoproteins essential to the functions as redox enzymes, such as glutathione peroxidase 4 (GPX4), a key player in ferroptosis.^[^ ^1^ ^]^ Selenium and selenoproteins are fundamental for various physiological processes and are implicated in numerous diseases, including neurodegenerative disorders,^[^ ^2^ ^]^ cancer,^[^ ^3^ ^]^ cardiovascular diseases,^[^ ^4^ ^]^ and diabetes.^[^ ^5^ ^]^ Understanding the biology of selenoproteins is therefore critical for elucidating the molecular mechanisms underlying these conditions.

Selenoprotein biosynthesis involves a unique and tightly regulated mechanism where the UGA codon, typically a stop codon, is repurposed to encode Sec during the elongation of the polypeptide chain. This process requires the interaction of the selenocysteine insertion sequence‐binding protein 2 (SECISBP2) with the selenocysteine insertion sequence (SECIS) element in the 3′ untranslated region (3′ UTR) of mRNAs. This interaction recruits the Sec‐specific elongation factor eEFSec and Sec‐tRNA^Sec^, enabling Sec incorporation at UGA codons.^[^ ^6^ ^]^ To date, through mining SEICS, 24 and 25 selenoproteins have been identified in mice and humans, respectively.

Despite their importance, mapping known selenoproteins and identifying new ones remain challenging due to the rarity of Sec in proteomes, substantial interference from Cys in thiol‐based labeling, and the incomplete coverage of existing selenoprotein databases. To address these issues, we previously developed a mass spectrometry‐based approach termed SecMS (selenocysteine‐specific mass spectrometry).^[^ ^7^ ^]^ This method uses the lower *pKa* of Sec (≈5.3) compared to Cys (≈8.5), enabling the selective labeling of Sec with a biotinylated electrophilic probe (EZ‐Link Iodoacetyl‐PEG2‐Biotin, IodoAPB) under acidic conditions, where Cys remains protonated and unreactive. SecMS facilitated the specific enrichment and identification of nearly the entire mouse selenoproteome, uncovering 22 of 24 known selenoproteins.^[^ ^7^ ^]^ Furthermore, we developed the SECIS‐Independent Selenoprotein (SIS) database, which translates all UGA stop codons into Sec, making the database searching based identification possible for exploring new selenoproteions. Combining SecMS with the SIS database allowed us to identify at least five previously unknown selenoproteins.^[^ ^7^ ^]^

Data‐independent acquisition (DIA) mass spectrometry has recently gained attention for its ability to provide comprehensive data collection, along with accurate and reproducible quantification.^[^ ^8^ ^]^ Conventional DIA analyses often require spectral libraries generated through data‐dependent acquisition (DDA) experiments, which can be time‐consuming and incomplete.^[^ ^9^ ^]^ More recently, advances in deep learning have enabled the prediction of in silico spectral libraries, providing a DDA‐free, accurate, and comprehensive alternative for both regular and modified peptides.^[^ ^10^ ^]^ These algorithms, such as DeepRT,^[^ ^11^ ^]^ Prosit,^[^ ^12^ ^]^ pDeep,^[^ ^13^ ^]^ DeepMass,^[^ ^14^ ^]^ DeepDIA,^[^ ^15^ ^]^ Predfull,^[^ ^16^ ^]^ DeepPhospho,^[^ ^17^ ^]^ DeepGP,^[^ ^18^ ^]^ and AlphaPeptDeep,^[^ ^19^ ^]^ have achieved remarkable success in predicting retention times, MS/MS, and ion mobilities.

The prediction‐based strategy is extremely appealing, especially for identifying novel selenopeptides that have never been captured through DDA analysis. However, deep learning‐based spectral prediction cannot be easily applied to selenopeptides due to the limited number of spectra for known selenopeptides, which severely hinders the accuracy of model training. To address this limitation, we developed DeepSecMS, where we leverage the similarities between Cys and Sec and utilize a large dataset of Cys‐containing peptides as proxies for model training. Under the AlphaPeptDeep framework,^[^ ^19^ ^]^ we generated an accurate model capable of predicting key features of both Sec and Cys peptides.

We demonstrate that by integrating SecMS, deep learning‐based selenopeptide library generation, and DIA, DeepSecMS significantly enhances the analysis of known selenoprotein identification and facilitates the discovery of several novel selenoprotein candidates in both cell and tissue samples. This innovative approach represents a significant advancement in selenoproteomics, offering new tools into selenoprotein exploration and its potential roles in human health and disease. More importantly, this proxy training strategy can be applied to other rare protein modifications. For instance, training on large‐scale acetylome data enables prediction of mass spectrometry data for other lysine acylation types.

## 2 Results and Discussion

### 2.1 Similarity between Sec‐ and Cys‐Substituted Peptide Pairs

Deep learning‐based peptide spectral prediction typically requires extensive datasets to train models effectively for accurate predictions. However, the scarcity of Sec‐containing peptides in existing datasets critically limits robust predictive model development for selenopeptides. To address this limitation, we investigated whether the abundant Cys‐containing peptides could serve as surrogates for Sec‐containing peptides in model training. Given the high similarity in physicochemical properties between Sec and Cys residues, this approach could potentially enable the prediction of both Cys‐ and Sec‐containing peptides.

**Figure 1:**
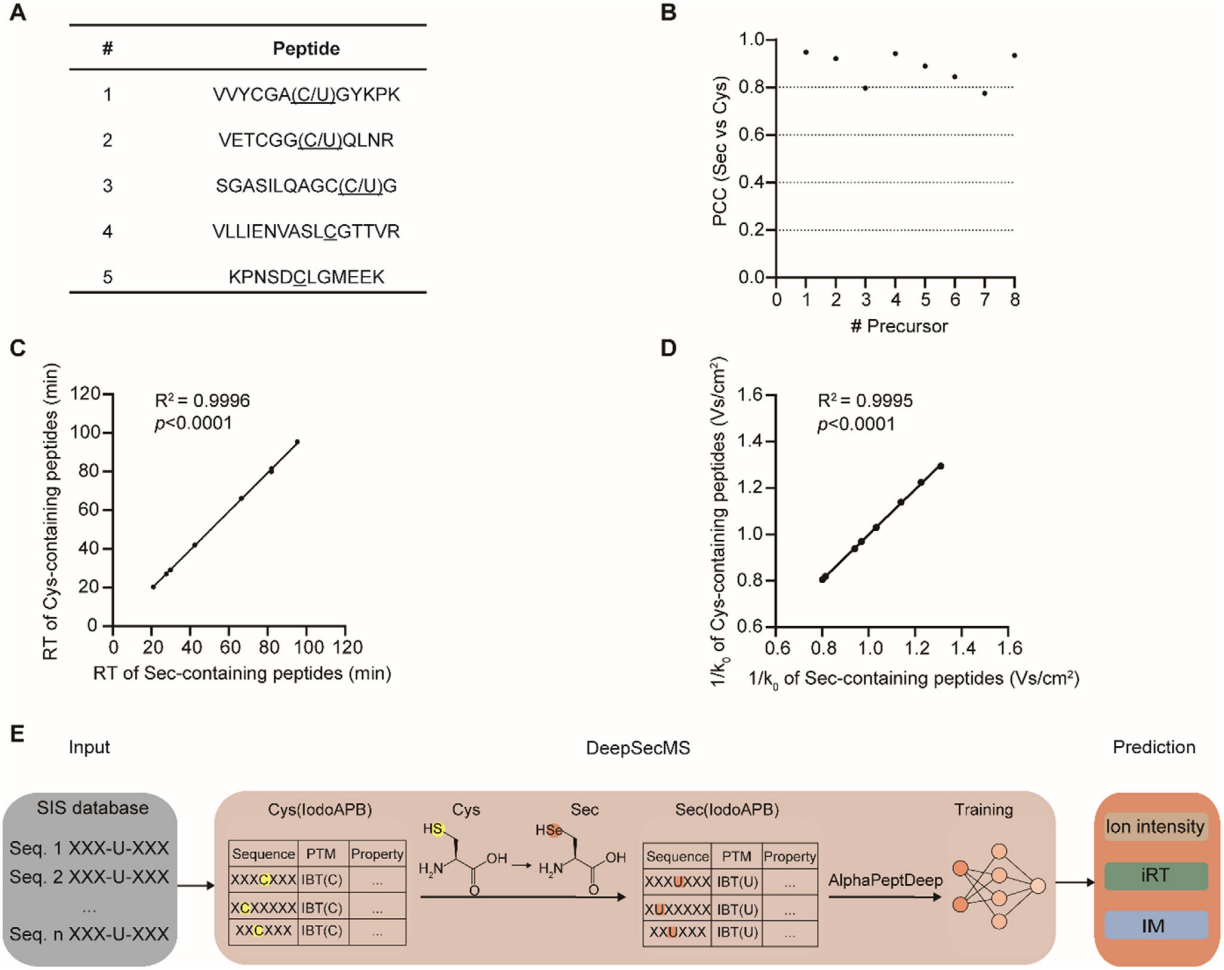
Similarity between Sec‐ and Cys‐substituted peptides and the DeepSecMS workflow. A) Five selected peptide pairs with Sec‐ and Cys‐substitution. B) Pearson correlation coefficient (PCC) of MS2 ion intensities between Sec‐ and Cys‐substituted peptides. C) Correlation of RT between Sec‐ and Cys‐ substituted peptides. Linear regression analysis was performed to assess this relationship, with the coefficient of determination (R^2^) shown along with the *p*‐value calculated using a two‐tailed test. D) Correlation of IM between Sec‐ and Cys‐ substituted peptides. Linear regression was used to calculate the R^2^ value, and statistical significance was evaluated using a two‐tailed test. E) DeepSecMS workflow.

To validate this hypothesis, we assessed the similarity between five paired peptides with Sec and Cys substitutions. The first three peptide pairs consisted of all synthetic peptides, with sequences derived from mouse selenoprotein W (SELENOW), human selenoprotein M (SELENOM), and thioredoxin reductase 1 (TXNRD1). The peptides VLLIENVASLCGTTVR and KPNSDCLGMEEK were synthetic, while their endogenous Sec‐containing counterparts originated from glutathione peroxidase 1 (GPX1) and selenoprotein I (SELENOI) in HEK 293T cells (**Figure** **1**A). These synthetic peptides were spiked into Sec‐enriched HEK 293T cell samples prepared using a selective Sec labeling strategy under low pH conditions.^[^ ^7^ ^]^ We then compared the MS2 (Figure 1B), retention time (RT) (Figure 1C), and ion mobility (IM) (Figure 1D) of the five peptide pairs. Our results demonstrated a high degree of similarity between the paired Sec‐ and Cys‐containing peptides, confirming that Cys‐containing peptides can serve as valid proxies for Sec‐containing peptides in training deep learning models.

### 2.2 Experimental Scheme of DeepSecMS

We constructed DeepSecMS based on the AlphaPeptDeep framework.^[^ ^19^ ^]^ (Figure 1E) In the SecMS strategy, Sec residues are labeled using the chemical probe IodoAPB.^[^ ^7^ ^]^ To leverage this approach, we first used the same probe to label Cys residues, generating a large spectral library of 34,996 Cys‐containing peptides from HEK 293T cells. We then intentionally substituted the Cys residues with Sec in these peptides, and adjusted the masses for both parent and product ions that originally contain Cys residues in the library. This acquired pseudo‐Sec spectral library was then used to train the predictive model. Subsequently, the model was applied to predict Sec‐containing peptides from SIS databases, resulting in MS2, RT, and IM data for 198,351 and 197,328 Sec‐containing peptides for human and mouse, respectively. Collectively, we developed DeepSecMS, a novel deep learning‐based method to generate a comprehensive predicted spectral library covering all potential Sec‐containing peptides in the SIS database. This strategy will enhance Sec‐containing peptide detection for both known and yet‐to‐be‐identified selenoproteins.

### 2.3 Evaluation of DeepSecMS for MS/MS Prediction

**Figure 2:**
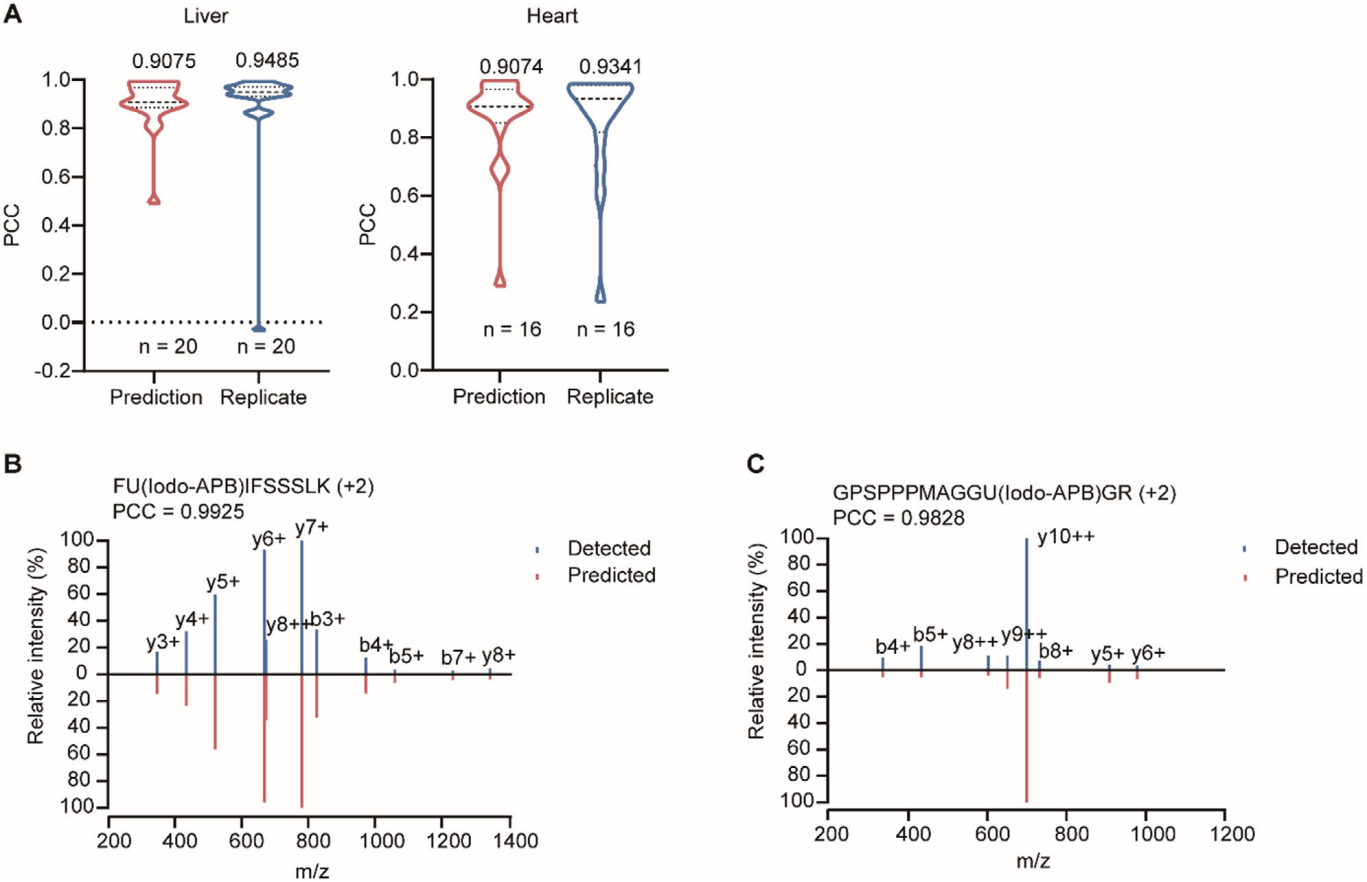
Evaluation of DeepSecMS for MS/MS prediction. A) High similarity between predicted and experimental MS2 for Sec‐containing peptides. The MS2 similarity, represented by the PCC values, is shown for comparisons between predicted and detected spectra, as well as between replicates. “n” indicates the number of spectra used for comparison. B,C) Representative predicted and detected MS2 spectra. Selenopeptides from mouse MSRB1 (B) and human SELENOK (C) are shown.

To evaluate the accuracy of the spectral library predicted by DeepSecMS, we compared the predicted spectra with those from a project‐specific DDA library for known selenoproteins, which was constructed using SecMS data from mouse liver and heart tissues with four biological replicates each. We found that the similarity between the predicted spectra and the experimental spectra for these selenopeptides was extremely high. The Pearson correlation coefficient (PCC) median values were 0.9075 and 0.9074 for these two datasets (**Figure** **2**A). These values were quite close to the similarity observed between two biological replicates of these tissues, which had median PCC values of 0.9485 and 0.9341, respectively (Figure 2A). We further show spectral comparisons for selenopeptides derived from mouse methionine‐R‐sulfoxide reductase B1 (MSRB1) (Figure 2B) and human selenoprotein K (SELENOK) (Figure 2C). These results demonstrate that the predicted and experimental spectra are highly comparable, confirming the accuracy of DeepSecMS in MS2 spectral prediction. Overall, DeepSecMS performs excellently in predicting spectra, highlighting its utility for accurate spectral prediction in selenoproteomics.

### 2.4 Evaluation of DeepSecMS for iRT and IM Prediction

**Figure 3:**
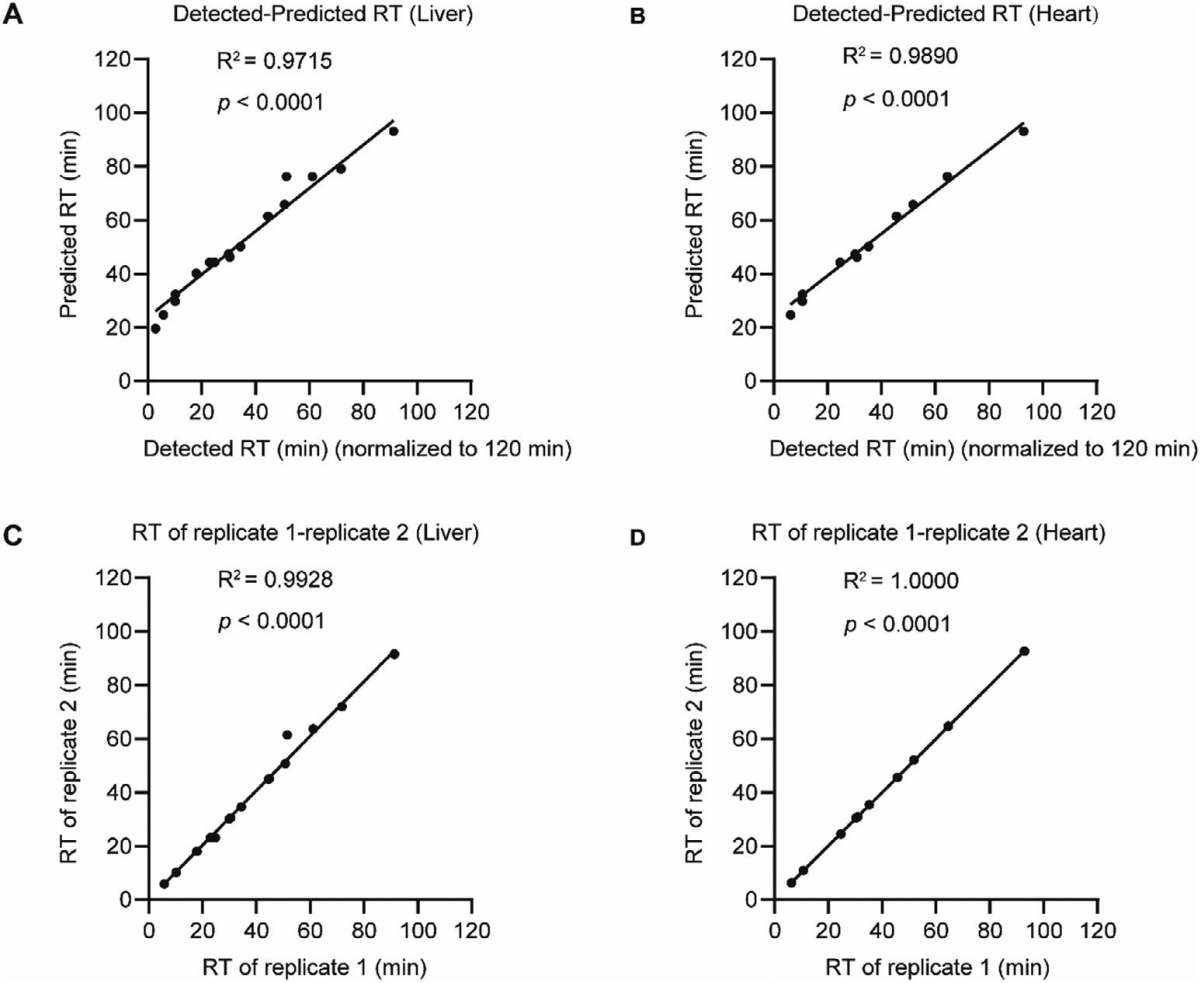
Evaluation of DeepSecMS for iRT prediction. A,B) Correlation between predicted and experimental iRT for Sec‐containing peptides. In the liver dataset, 20 Sec‐containing peptides were analyzed, while in the heart dataset, 16 Sec‐containing peptides were included. The detected RTs were normalized using EasyPQP within FragPipe by scaling the experimental RTs to a standard 120 min gradient. C‐D) Correlation of iRT values between replicates for Sec‐containing peptides.

**Figure 4:**
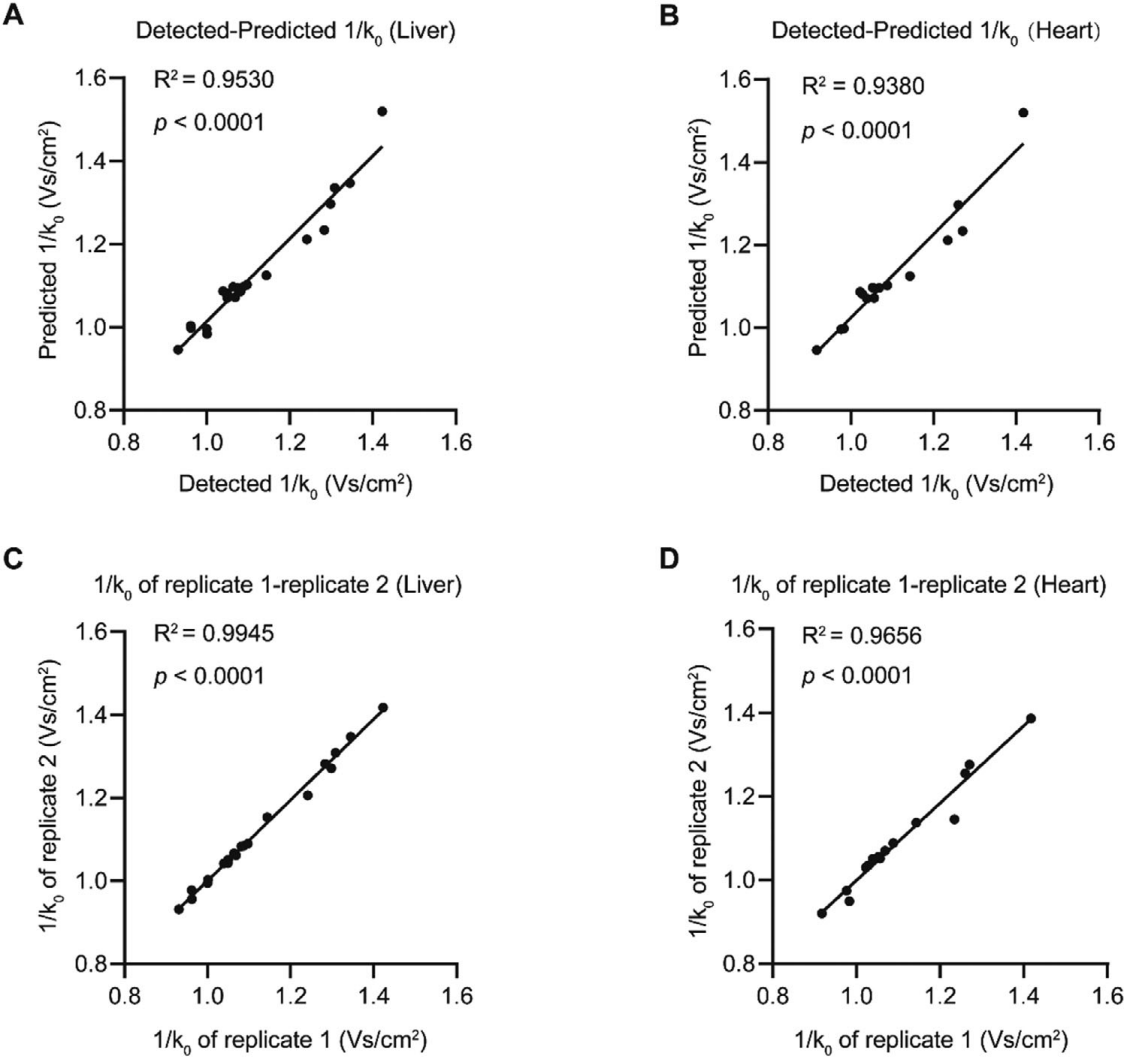
Evaluation of DeepSecMS for IM prediction. A,B) Correlation between predicted and experimental IM values for Sec‐containing peptides. In the liver dataset, 20 Sec‐containing peptides were analyzed, while in the heart dataset, 16 Sec‐containing peptides were included. C,D) Correlation of IM values between replicates for Sec‐containing peptides.

Next, we evaluated the performance of DeepSecMS in predicting indexed retention times (iRT) and IM of Sec‐containing peptides by comparing its predictions with experimental spectral libraries generated using SecMS DDA data. The predicted iRT values showed a highly significant correlation with the experimental libraries, with R^2^ values of 0.9715 for liver (**Figure** **3**A) and 0.9890 for heart (Figure 3B). These values closely approached the correlations observed between biological replicates, which had R^2^ values of 0.9928 for liver (Figure 3C) and 1.0000 for heart (Figure 3D). In addition, the IM predictions for Sec‐containing peptides demonstrated strong accuracy, with R^2^ values of 0.9530 (**Figure** **4**A) and 0.9380 (Figure 4B). The correlations are comparable to those observed between biological replicates (Figure 4C,D). These results underscore the remarkable accuracy of DeepSecMS in predicting both iRT and IM values for Sec‐containing peptides, further highlighting its potential as a reliable tool in selenoproteomics.

### 2.5 DeepSecMS Library Optimization

The DeepSecMS‐predicted library includes all tryptic peptides containing Sec derived from the SIS database. This library contains an extensive set of 507,747 precursors, generated using the following criteria: charge states of +1, +2, +3, and +4, fragment ions in +1 and +2 charge states, and retention of all fragment types (b and y ions). However, most peptides in this library are hypothetical and not biologically present, with only a tiny fraction being real (e.g., in the worst‐case scenario, only 25 selenoproteins are genuinely present in the human SIS database). To address this limitation and prevent the DIA identification scoring system from becoming unreliable due to the library being predominantly composed of hypothetical, non‐existing peptides, we optimized the library in two ways.

First, we refined the Sec‐containing peptides in the SIS database by filtering them based on the binding ability of their encoding RNAs to SECISBP2, a key RNA‐binding protein in the Sec insertion machinery.^[^ ^20^ ^]^ We refer to this set as Selenoprotein Consensus from SECISBP2‐Seq and SIS (SCoSS), representing high‐confidence candidates supported by both RNA‐level SECISBP2 binding and the presence of in‐frame UGA codons, thereby increasing their potential to encode functional selenoproteins. The SCoSS library comprises 986 potential selenoproteins (Table S1, Supporting Information) and 11,551 precursors. Importantly, this trimmed library retains 24 known human selenoproteins, excluding TXNRD3, whose tryptic peptide sequence (GCUG) is too short to be reliably detected by mass spectrometry.

**Figure 5:**
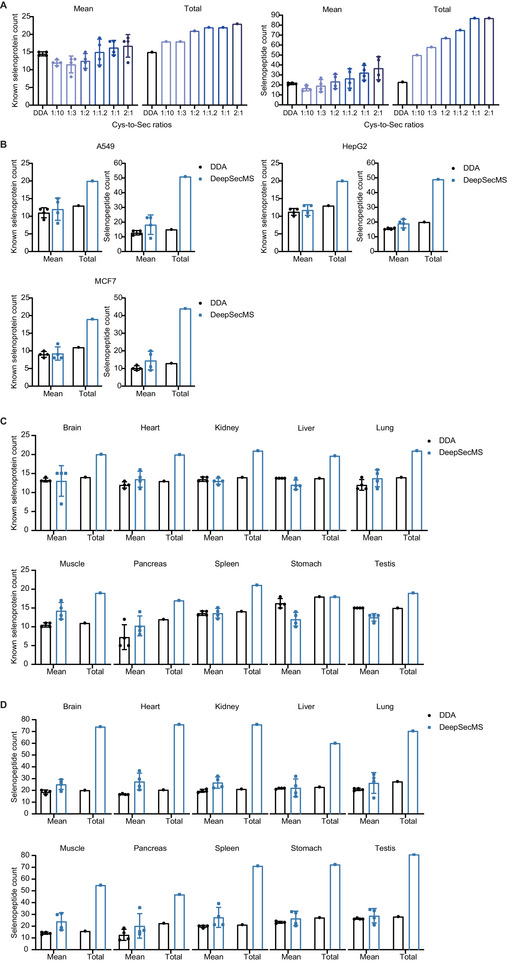
DeepSecMS enhances the identification of selenoproteins. A) The predicted SCoSS library with buffering peptide enhances selenoprotein identification. “Mean” values reflect the identification results obtained from four individual biological replicates, while “Total” represents the cumulative number of identified entities across all four replicates. Peptides sharing an identical amino acid sequence but bearing distinct modifications are each counted as separate selenopeptide entries. B) DeepSecMS improves selenoprotein identification across multiple cell lines. C,D) Mouse tissue‐specific selenoproteome maps acquired using DeepSecMS. DeepSecMS enhances the identification of both selenoproteins (C) and Sec‐containing peptides (D).

Second, we incorporated buffering peptides (Table S2, Supporting Information) into the SCoSS library. These buffering peptides were those Cys‐containing peptides that are detected with high intensities in our SecMS analysis. The Cys‐to‐Sec ratios in the hybrid library were 1:10, 1:3, 1:2, 1:1.2, 1:1, and 2:1. Using these combined libraries (Table S3, Supporting Information) to analyze the DIA data from HEK 293T cells, we demonstrated that the inclusion of Cys‐containing buffering peptides significantly improved the identification of both selenoproteins and selenopeptides (**Figure** **5**A; Figure S1A and Table S4, Supporting Information), highlighting the critical role of buffering peptides in enhancing the performance of DeepSecMS for selenoprotein analysis. Specifically, at a buffering ratio ranging from 1:2 to 2:1 (Cys:Sec), we successfully identified 21–23 unique selenoproteins, nearly reaching the theoretical maximum and surpassing the 15 identified from the DDA data. We therefore chose a buffering ratio of 2:1 (Cys:Sec) for the subsequent analysis.

### 2.6 DeepSecMS Enhances the Identification of Selenoproteins

To systematically compare the DeepSecMS‐DIA approach with the conventional DDA method for selenoprotein identification, we analyzed SecMS samples from various cell lines, including A549, HepG2, and MCF7, in both DDA and DIA modes using a timsTOF Pro 2 (Bruker) mass spectrometer. The results consistently demonstrated that the DeepSecMS predicted SCoSS library significantly enhanced the identification of known selenoproteins at both the protein and peptide levels across these cell lines (Figure 5B; Figure S1B and Table S4, Supporting Information). This improvement was particularly evident in the total number of identified selenoproteins when combining identifications from biological replicates. These results strongly support that DeepSecMS is a robust tool for identifying selenoproteins.

### 2.7 DeepSecMS Profiles Tissue‐specific Selenoproteomes in Mice

Furthermore, using DeepSecMS, we analyzed DIA SecMS data collected from ten distinct mouse tissues, including brain, kidney, liver, lung, muscle, spleen, heart, pancreas, stomach, and testis (Table S5, Supporting Information). The results consistently demonstrated that DeepSecMS significantly enhances the identification of known selenoproteins (Figure 5C; Figure S1C and Table S6, Supporting Information) and IodoAPB‐labeled selenopeptides (Figure 5D; Table S6, Supporting Information). This represents a significant advancement compared to the first selenoproteome map obtained years ago using DDA.^[^ ^7^ ^]^

Overall, our analysis across diverse biological samples highlights that the DeepSecMS predicted spectral library substantially enhances the depth of known selenoprotein identification, further confirming the accuracy and efficacy of this strategy. Moreover, the proxy training strategy developed here could inspire approaches for mapping other rare post‐translational modifications, broadening the applicability of data‐ and deep learning‐ driven proteomics. For instance, large‐scale lysine acetylome data can be employed to train models for other rare lysine acyl modification profiling.

### 2.8 DeepSecMS Identifies Novel Selenoprotein Candidates

**Figure 6:**
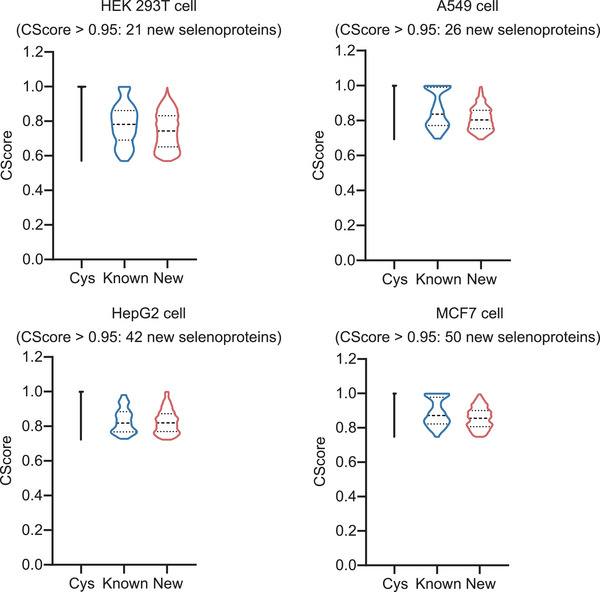
DeepSecMS facilitates the discovery of novel selenoprotein candidates. DeepSecMS enables the identification of novel selenoproteins with high Cscore across various cell lines.

The inclusion of all theoretical Sec‐containing peptides in the SIS database facilitated our previous identification and validation of five novel selenoproteins from mouse tissues using SecMS DDA data.^[^ ^7^ ^]^ Building upon this data, we utilized DeepSecMS for a more in‐depth exploration of the selenoproteome, using SecMS DIA data derived from various cell types, including HEK 293T, A549, HepG2, and MCF7 cells. Our findings demonstrate that the DeepSecMS‐predicted SCoSS spectral library significantly improves the identification of known selenoproteins, while also enabling the identification of several novel selenoproteins (**Figure** **6**). Specifically, 21, 26, 42, and 50 new selenoproteins were identified from these four cell lines (Table S4, Supporting Information). The identification confidence scores (Cscore)^[^ ^21^ ^]^ for these novel selenoproteins are comparable to those of known selenoproteins, suggesting the potential for validating these newly discovered selenoproteins. Though extremely challenging, our results raise the possibility of validating these novel selenoproteins, highlighting the substantial potential of the predicted spectral library for discovering new selenoproteins in proteomics research. Crucially, the proxy training strategy could be adapted to systematically identify novel proteins harboring other rare post‐translational modifications or amino acid substitution, where conventional discovery pipelines are hindered by insufficient reference data.

## 3 Conclusion

In this study, we employed a Cys‐to‐Sec proxy training strategy to establish the deep‐learning‐based DeepSecMS for the analysis of rare selenoproteomes. Using DeepSecMS, we demonstrated its ability to significantly enhance the identification of known selenoproteins, improve tissue‐specific selenoproteome mapping, and facilitate the discovery of novel selenoprotein candidates. More importantly, these findings highlight the powerful potential of DeepSecMS and the proxy training strategy to advance the analysis of other rare protein variants and modifications.

## 4 Experimental Section

### Sample Preparation

For the preparation of synthetic standard peptides, 1 mg of lyophilized powder was dissolved in either H_2_O or DMSO, based on its solubility. 40 µg of each peptide was used for selenoprotein enrichment. Human cell lines, including HEK 293T, A549, HepG2, and MCF7, were harvested by washing three times with pre‐chilled PBS to remove residual culture media. Cells were lysed on ice in 8 m urea solution for ≈10 min. The lysis buffer contained 8 m urea (Sigma–Aldrich, catalog: U5128), 100 mM Tris (pH 8.5) (Sigma–Aldrich, catalog: T1503), and a protease inhibitor cocktail (Roche, catalog: 11836145001). The cell lysates were sonicated for 30 s and centrifuged at 20,000 g for 10 min at 4 °C to obtain the supernatant. Protein concentration was measured using a BCA assay (Yeasen, catalog: 20201ES90), and 2 mg of protein per sample was reserved for enrichment.

Mouse tissue samples were obtained from four male C57BL/6 mice. The experimental protocols involving animals were authorized by the Institutional Animal Care and Use Committee of the Chinese Academy of Sciences after rigorous evaluation, strictly following the Guide for the Care and Use of Laboratory Animals of Chinese Academy of Sciences. For each tissue type, a specific weight of the sample was selected, and residual blood was removed using pre‐chilled PBS. The samples were then placed into homogenization tubes (2 mL PP microtubes) along with an appropriate amount of 0.5 mm glass beads and 1 mm ceramic beads, in addition to the 8 m urea solution. A multifunctional sample homogenizer and a cryogenic grinder were utilized to homogenize the tissues until no visible fragments remained. Following this, the samples were centrifuged at maximum speed for 10 min at 4 °C, and the supernatant was collected for BCA assay to measure protein concentration. Again, 2 mg of protein from each sample was retained for subsequent enrichment operations.

The enrichment of selenoproteins was carried out as described previously.^[^ ^6^, ^7^ ^]^ Briefly, samples were incubated with a final concentration of 20 mM DTT (Sigma–Aldrich, catalog: 43819) at 37 °C for 30 min. Subsequently, an equal volume of citric acid solution was added to adjust the pH of the samples to ≈4.0. In the absence of light, IodoAPB (Thermo Fisher Scientific, catalog: 21334) was added to a final concentration of 0.1 mM, and the samples were incubated at 25 °C for 60 min to allow for the specific reaction of Sec with IodoAPB, thus labeling the Sec with biotin. Excess IodoAPB was removed using a 30‐kDa centrifugal filter device (Merck, catalog: UFC5030BK), and the solution was replaced with 8 m urea buffer (pH 8.5). Concurrently, iodoacetic acid (IAA) (Sigma–Aldrich, catalog: I1149) was introduced to a final concentration of 5 mM to facilitate alkylation of Cys residues. Once the labeling reaction was complete, the enriched samples proceeded to the next step.

For both cell and tissue samples, the protein solution retained in the 30‐kDa centrifugal filter device was combined with 200 µL of 100 mM Tris (pH 8.5) until complete solubilization of the protein. A final concentration of 1 mM CaCl₂ solution was then added. Trypsin was introduced at a mass ratio of 1:100 (trypsin to protein) for an overnight digestion at 37 °C with shaking. Following digestion, the peptide mixture was subjected to enrichment of biotin‐labeled Sec‐containing peptides using a C8 column filled with streptavidin. After loading the digested peptides onto the C8 column, the column was washed three times sequentially with 200 µL of PBSN, 200 µL of PBS, and 200 µL of 10% acetonitrile (ACN). Finally, biotin‐labeled peptides were eluted with 100 µL of 50% ACN and 2% trifluoroacetic acid (TFA). The eluate was concentrated in a vacuum concentrator at 4 °C for 2 h. The dried samples were then resuspended in 20 µL of 0.1% formic acid (FA) prior to LC‐MS/MS analysis.

### LC‐MS/MS Analysis

The LC‐MS/MS analysis was performed using a timsTOF Pro 2 (Bruker) mass spectrometer, coupled with a nanoElute liquid chromatography platform. 25 cm capillary column (75 µm I.D.) with an ESI emitter tip is laser‐pulled and packed in house with C18 resin (C18‐AQ, 1.5 µm) (Dr. Maisch, Germany). The mobile phases consisted of solvent A (80% H_2_O, 20% ACN, and 0.1% FA) and solvent B (80% ACN, 2% H_2_O, and 0.1% FA). Each sample was subjected to a 30 min elution at a flow rate of 300 nL min^−1^, with the column oven maintained at 50 °C. The elution gradient was as follows: 5% to 10% B for 2 min; 10% to 35% B for 21 min; 35% to 85% B for 1 min; 85% to 95% B for 1 min; 95% to 98% B for 2 min; and finally, 98% B for 3 min.

Two data acquisition modes were employed: PASEF and dia‐PASEF. In the PASEF mode, the ramp time was set to 100 ms, with a total cycle time of 1.17 s. During each topN cycle, 10 PASEF scans were conducted, covering an m/z range from 100 to 1700, and accommodating ion charge states from +1 to +5. The ion mobility range was set between 0.6 and 1.6 Vs cm^−^^2^, with an automatic gain control (AGC) target of 20 000 and a dynamic exclusion time of 0.4 min, corresponding to a mass range of 0.015 m/z and 1/K_0_ of 0.015 Vs cm^−^^2^. The quadrupole separation width was dependent on the m/z ratio: set to 2 m/z for m/z < 700, 3 m/z for m/z > 800, and varying linearly for 700 < m/z < 800. Collision energy was dynamically adjusted based on ion mobility; maintained at 20 eV when 1/K_0_ was below 0.6 Vs cm^−^^2^ and at 59 eV when above 1.6 Vs cm^−^^2^, with a linear gradient in between. For the dia‐PASEF mode, the following parameters were utilized: ramp time of 100 ms; full scan range from 350 to 1400 m/z; accommodating charge states from unassigned (0) to +5; and ion mobility set at 0.75 to 1.4 Vs cm^−^^2^. The AGC target was set at 10 000, with a dynamic exclusion time of 0.5 min (corresponding to a mass range of 0.015 m/z and 1/K₀ of 0.015 Vs cm^−^^2^). The separation window width was fixed at 30 m/z, encompassing a range of 375–1380 m/z.

### Construction of DeepSecMS Predicted Spectral Library

The predictive spectral library for selenoproteins was constructed based on the structural similarity between Sec and Cys. DeepSecMS was fine‐tuned based on AlphaPeptDeep's pre‐trained models,^[^ ^19^ ^]^ and the model architecture and training parameters were provided in **Table** **1**. Utilizing the DeepSecMS model, we predicted the spectra of Sec‐containing peptides from the SIS database. The training dataset comprised a comprehensive spectral library of Cys‐containing peptides previously generated by the research group, which included 34,996 labeled peptides derived from IodoAPB‐labelled proteins in HEK 293T cells. Given the limited number of known Sec‐containing peptides available for training prediction models, mass differences between Se and S in the molecular structures of Sec and Cys were capitalized. Initially, Cys residues in the training spectral library were transformed to their corresponding Sec forms through a “Cys‐S+Se” conversion process. This conversion enabled the generation of an expanded spectral library representative of Sec‐containing peptides. Subsequently, this transformed Sec spectral library was employed to train the AlphaPeptDeep model. The prediction focused on all Sec‐containing peptides within the SIS database.^[^ ^7^ ^]^

**Table 1:** Model architecture and details for DeepSecMS.

Model	Component	Details
MS2	Amino Acid Embedding	AlphaPeptDeep uses torch.nn.Embedding for amino acid ASCII codes
	PTM Embedding	AlphaPeptDeep's built‐in chemical composition count encoding followed by a FC layer
	Meta Embedding	Precursor charges, NCEs, Instrument types are considered in AlphaPeptDeep
	Transformer Layers	4 layers, dim = 256
	Fully Connected (FC) Layers	2 layers, dim = 128, dim = 8 (output)
	Number of Trainable Parameters	3988974
	Pretrained Model	The pretrained “generic” models were pre‐loaded for fine‐tuning, see https://alphapeptdeep.readthedocs.io/en/latest/module_pretrained_models.html#peptdeep.pretrained_models.ModelManager.load_installed_models
RT/CCS	Amino Acid Embedding	One‐hot encoder
	PTM Embedding	AlphaPeptDeep's built‐in chemical composition count encoding
	Meta Embedding	Precursor charge encoding for CCS model
	1D‐CNN layer	1 layer, channel number = 128
	LSTM‐layer	2 layers, dim = 128
	FC layers	2 layers, dim = 64 and dim = 1 (output)
	Number of Trainable Parameters	RT = 708,224; CCS = 713,452
	Pretrained Model	The pretrained “generic” models were used for fine‐tuning, see https://alphapeptdeep.readthedocs.io/en/latest/module_pretrained_models.html#peptdeep.pretrained_models.ModelManager.load_installed_models
Fine‐tuning	Loss Function	L1 Loss
	Fine‐tuning Epochs	20
	Learning Rate Scheduler	Cosine scheduler with warm‐up (HuggingFace implementation)
	Warm‐up Epochs	5
	Learning Rate	1 × 10⁻⁵
	Dropout Rate	0.1
	Mini‐Batch Size	256

### Project‐Specific DDA Library Generation

The project‐specific DDA library was generated using the FragPipe software (version 20.0),^[^ ^22^ ^]^ employing the workflow “DIA_SpecLib_Quant.” The following parameters were configured: the mass spectrometry data type was set to IM‐MS, and the database selection included either the human or mouse SIS database, supplemented with decoy and contaminant proteins. DDA data search was conducted using MSFragger with the following settings: the search tolerance for precursor and fragment ions was established at 20 ppm, and both mass calibration and parameter optimization were enabled. Trypsin was selected as the enzyme, allowing for a maximum of two missed cleavages. The peptide length was constrained to 7–50 amino acids, with a mass range of 500–5000 Da. Variable modifications included oxidation of methionine (+15.9949 Da), acetylation of N‐terminal proteins (+42.0106 Da), carbamidomethyl of cysteine and selenocysteine (+57.02 1464 Da), biotin‐labeled carbamidomethyl of cysteine and selenocysteine (+414.193 691 Da), and deselenization of selenocysteine (‐81.932 170 Da). The minimum number of matched fragment ions was set to 4, with a maximum fragment ion charge state of 2. In the validation module, MSBooster was employed to rescore the predicted RT and spectra. Error rates were estimated using Percolator,^[^ ^23^ ^]^ and protein inference was performed via ProteinProphet.^[^ ^24^ ^]^ The spectral library was generated using the built‐in EasyPQP tool, with automatic selection of a reference for RT and IM calibration. The RT Lowess score was set to 0. The mass tolerance for fragment ions was specified at 15 ppm and fragment ion types were designated as b and y ions.

### DIA Data Analysis

For the analysis of SecMS DIA data, DIANN (version 20.0)^[^ ^25^ ^]^ was utilized, incorporating stringent settings for optimized peptide and protein identification. The analysis was conducted using two spectral libraries: a project‐specific DDA library and the DeepSecMS predicted spectral library. The SIS sequence database was employed, which allowed for the reannotation of proteins based on this dataset. Both precursor and fragment mass accuracy were automatically adjusted by DIANN with the precision parameters set to 0, ensuring optimal alignment based on the data. The scan window was similarly left at 0, allowing for the software to determine the best configuration dynamically. Isotopic isomers were included in the analysis, and the Match Between Runs (MBR) feature was enabled to enhance the detection of peptides across different samples. For protein inference, a heuristic approach was selected, with inference conducted at the gene level. A neural network classifier was employed in single‐pass mode to enhance the accuracy of peptide identification. Quantification was performed using the robust LC strategy, known for its high precision, while cross‐sample normalization was dependent on RT. False discovery rate (FDR) control was applied, with the precursor ion FDR threshold set at 1%.

### Ethical Statement

All experimental procedures were approved by Chinese Academy of Sciences Institutional Animal Care and Use Committee.

## Conflict of Interest

The authors declare no conflict of interest.

## Author Contributions

Y.Z. conceived and supervised the project. C.S. conducted the majority of the experiments and analyzed the data. Y.Y., Y.Z., and L.Q. provided experimental materials and technical supports. W.Z. provided crucial support with AlphaPeptDeep. Y.Z. and C.S. wrote the manuscript with inputs from all authors.

## Supporting information



Supporting Information

Supplementary Table 1

Supplementary Table 2

Supplementary Table 3

Supplementary Table 4

Supplementary Table 5

Supplementary Table 6

## Data Availability

The MS data for this study have been deposited to the ProteomeXchange Consortium via the iProX partner repository with the dataset identifier PXD063654.
