# Supplementary material for: DeepSecMS Advances DIA‐Based Selenoproteome Profiling Through Cys‐to‐Sec Proxy Training
Source: Adv Sci (Weinh). 2025 Jul 22;12(38):e04109. doi: 10.1002/advs.202504109 (PMC12520545; doi:10.1002/advs.202504109)
Supplement: Supplementary file 1 — Supporting Information [file ADVS-12-e04109-s006.pdf]

ADVANCED SCIENCE
Open Access

Supporting Information

for *Adv. Sci.*, DOI 10.1002/advs.202504109

DeepSecMS Advances DIA-Based Selenoproteome Profiling Through Cys-to-Sec Proxy Training

*Chenfang Si, Yamei Yuan, Yu Zong, Liang Qiao, Wen-Feng Zeng and Yaoyang Zhang**

Supporting Information

DeepSecMS Advances DIA-based Selenoproteome Profiling through Cys-to-Sec Proxy Training

*Chenfang Si, Yamei Yuan, Yu Zong, Liang Qiao, Wen-Feng Zeng, Yaoyang Zhang**

File list

1. Figure S1: Venn diagrams showing the overlap of known selenoproteins identified by DDA and DeepSecMS analyses.
2. Table S1: Candidate selenoproteins in the refined SCoSS library.
3. Table S2: Cys-containing peptides included under different buffering ratios.
4. Table S3: Human spectral library used for DeepSecMS.
5. Table S4: Identification of selenoproteins in HEK293T, A549, HepG2, and MCF7 cells.
6. Table S5: Mouse spectral library used for DeepSecMS.
7. Table S6: Identification of selenoproteins in mouse tissues.

Figure S1

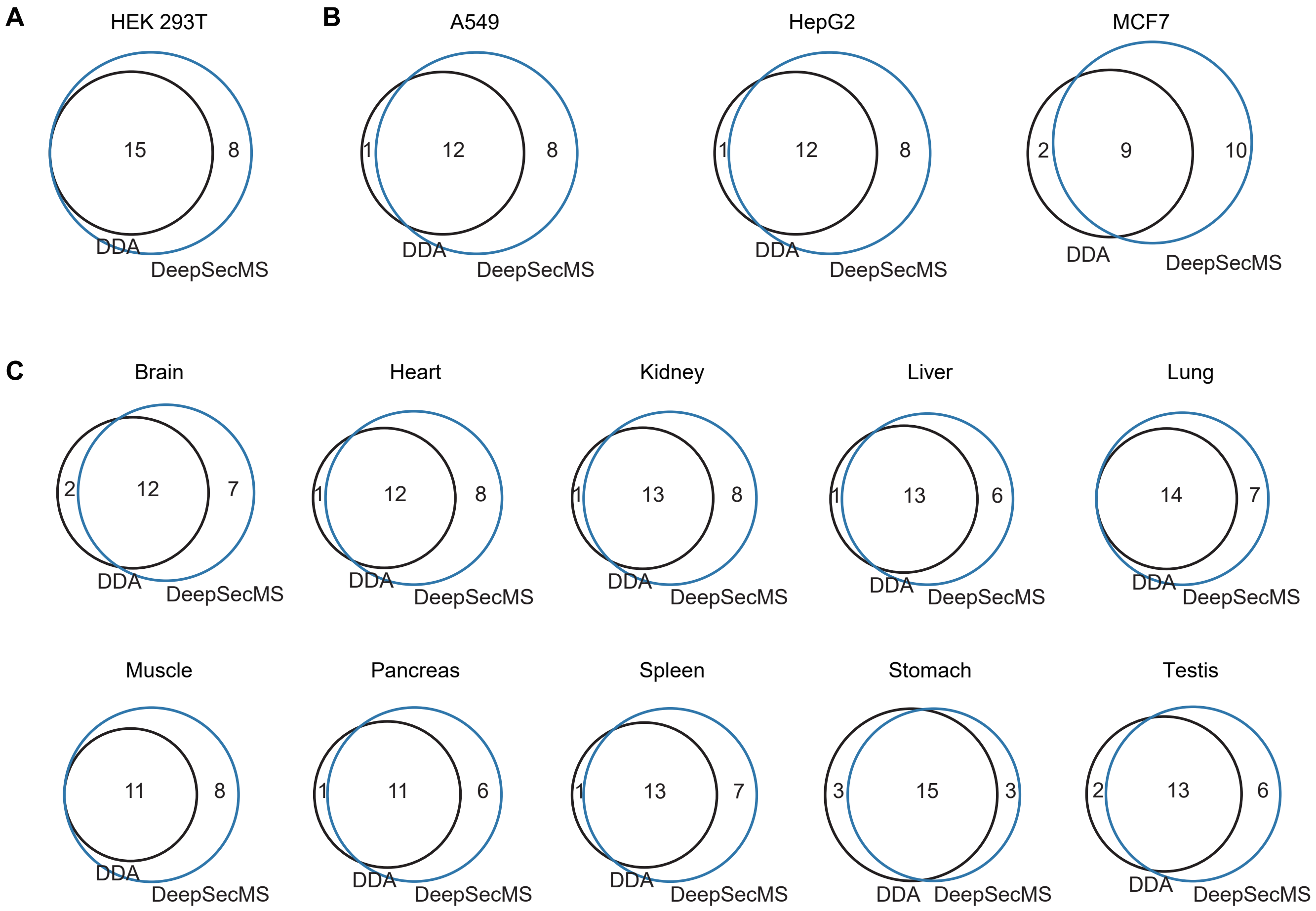

Figure S1. Venn diagrams showing the overlap of known selenoproteins identified by DDA and DeepSecMS analyses.
(A) Comparison of known selenoproteins identified by DDA and DeepSecMS in HEK 293T cells.
(B) Comparison of known selenoproteins identified by DDA and DeepSecMS in A549 cells, HepG2 cells, and MCF7 cells.
(C) Comparison of known selenoproteins identified by DDA and DeepSecMS across various mouse tissues.
